# Exopolysaccharide ID1 Improves Post-Warming Outcomes after Vitrification of In Vitro-Produced Bovine Embryos

**DOI:** 10.3390/ijms23137069

**Published:** 2022-06-25

**Authors:** Erika Alina Ordóñez-León, Iris Martínez-Rodero, Tania García-Martínez, Manel López-Béjar, Marc Yeste, Elena Mercade, Teresa Mogas

**Affiliations:** 1 Department of Animal Medicine and Surgery, Autonomous University of Barcelona, Cerdanyola del Vallès, ES-08193 Barcelona, Spain; alina.mvzalina@gmail.com (E.A.O.-L.); iris.martinez@outlook.com (I.M.-R.); taniagarciamartinez@gmail.com (T.G.-M.); 2 Brasuca In Vitro, Villahermosa MX-86040, Mexico; 3 Department of Animal Health and Anatomy, Autonomous University of Barcelona, Cerdanyola del Vallès, ES-08193 Barcelona, Spain; manel.lopez.bejar@uab.cat; 4 Department of Biology, Institute of Food and Agricultural Technology, University of Girona, ES-17004 Girona, Spain; marc.yeste@udg.edu; 5 Department of Biology, Health and Environment, University of Barcelona, ES-08007 Barcelona, Spain; mmercade@ub.edu

**Keywords:** cryopreservation, blastocyst, total cell number, inner cell mass, TUNEL, embryo development, gene expression regulation

## Abstract

This study aimed to assess the cryoprotectant role of exopolysaccharide (EPS) ID1, produced by Antarctic *Pseudomonas* sp., in the vitrification of in vitro-produced (IVP) bovine embryos. IVP day 7 (D7) and day 8 (D8) expanded blastocysts derived from cow or calf oocytes were vitrified without supplementation (EPS0) or supplemented with 10 µg/mL (EPS10) or 100 µg/mL (EPS100) EPS ID1. The effect of EPS ID1 was assessed in post-warming re-expansion and hatching rates, differential cell count, apoptosis rate, and gene expression. EPS100 re-expansion rates were significantly higher than those observed for the EPS0 and EPS10 treatments, regardless of culture length or oocyte source. EPS100 hatching rate was similar to the one of the fresh blastocysts except for those D7 blastocysts derived from calf oocytes. No differences were observed among EPS ID1 treatments when the inner cell mass, trophectoderm, and total cell number were assessed. Although apoptosis rates were higher (*p* ≤ 0.05) in vitrified groups compared to fresh embryos, EPS100 blastocysts had a lower number (*p* ≤ 0.05) of apoptotic nuclei than the EPS0 or EPS10 groups. No differences in the expression of *BCL2*, *AQP3*, *CX43,* and *SOD1* genes between treatments were observed. Vitrification without EPS ID1 supplementation produced blastocysts with significantly higher *BAX* gene expression, whereas treatment with 100 µg/mL EPS ID1 returned *BAX* levels to those observed in non-vitrified blastocysts. Our results suggest that 100 µg/mL EPS ID1 added to the vitrification media is beneficial for embryo cryopreservation because it results in higher re-expansion and hatching ability and it positively modulates apoptosis.

## 1. Introduction

In the recent decade, in vitro fertilization has developed as a viable alternative to superovulation and has become the preferred method for producing bovine embryos, particularly in zebu breeds. The increasing demand for in vitro-produced (IVP) bovine embryos is reflected in the last annual report by the International Embryo Transfer Society (IETS): Since 2016, the proportion of IVP embryos has been increasing, reaching 81.5% of all cattle transferrable embryos in 2020, at the expense of in vivo-derived embryos (IVD) [1]. The advantages of ovum pick-up (i.e., multiple donor types, a greater frequency of oocyte collection) combined with in vitro fertilization (i.e., smaller number of sperm required, use of different males, use of sexed semen) and genomic selection, have resulted in the widespread acceptance of IVP, contributing to an accelerated genetic gain in dairy breeds and an improvement in beef cattle markets [2]. Nevertheless, IVP must be combined with a reliable embryo freezing system that ensures the best use of the supernumerary embryos to allow the commercialization of the resulting superior genetics while assuring biosafety and quality [3,4]. While 39.5% of IVP embryos transferred in 2020 were frozen/thawed, this percentage is still below the 59.5% of transfers of frozen/thawed IVD embryos [1]. This difference owes to the lower cryotolerance of IVP embryos compared to their in-vivo counterparts [2,4]. Thus, despite the clear advantages of the IVP system in the cattle industry, it still faces the challenge of reducing the pregnancy rate gap between frozen IVP and fresh embryos to the same level as the gap between frozen IVD and fresh embryos [2].

Slow freezing and vitrification are the two common methods for cryopreservation of each IVD and IVP bovine embryos. Whereas both procedures could end in reduced embryo quality and a compromised pregnancy rate after transfer, vitrification appears as a superior procedure to slow freezing for cryopreserving IVP bovine embryos, which are more sensitive to cryoinjury [2,3,4]. Vitrification is a process during which solidification of a solution occurs without the formation of ice crystals. This phenomenon involves both the use of concentrated cryoprotectants (CPA) solutions and rapid cooling and warming rates. CPAs are classified according to their permeability to the cell membrane in: (1) permeating CPAs, commonly organic compounds characterized by a low molecular weight that move across the plasma membrane, form hydrogen bonds with intracellular water molecules, and avoid ice formation; and (2) non-permeating CPAs, high molecular weight compounds usually highly hydrophilic, that remain in the cryopreservation medium, protecting cells from high osmotic stresses and conferring less toxicity. Because non-permeating CPAs mimic the effects of intracellular solutes in the extracellular space, they allow that a lower concentration of the penetrating CPAs is needed for vitrification [5].

Several examples of biological functions similar to those played by non-permeating CPAs can be found in nature: species that live in freezing temperatures produce a wide variety of substances that protect them against low temperatures [6]. Among others, antifreeze proteins (AFPs) and glycoproteins (AFGPs), biosynthesized by some bacteria, fungi, microalgae, crustaceans, fish, and insects, facilitate cell survival at sub-zero temperatures by thermal hysteresis, ice recrystallization inhibition, and stabilization of cell membranes [7]. Because these cryoprotectant properties are interesting and could be incorporated into cryopreservation protocols, multiple attempts of supplementing cryopreservation media with AFPs and AFGPs have been made in oocytes [8,9,10,11,12,13] and embryos [14,15,16,17,18,19,20,21] of many species, leading to an improvement in survival rates.

Aside from AFPs, extracellular substances, composed primarily of exopolysaccharides (EPS), have reached a prominent position among non-permeable CPAs [22]. EPSs are sugar-based polymers with high molecular weight (10–30 kDa) synthesized and secreted by microorganisms. These polymers influence the physicochemical environment of cells and are believed to contribute to numerous processes involved in microbial cold adaptations [22,23]. The role of EPS in protecting microorganisms from cold-induced damage caused by freezing conditions in cold and icy environments has been already reported [23,24,25]. Besides, previous studies showed that not only does the cryoprotective activity of EPS benefit the cold-adapted bacterial producer, but also non-EPS-producing cells, suggesting a universal cryoprotectant role for these biopolymers [24,25,26].

Exopolysaccharide ID1 (EPS ID1) produced by *Pseudomonas* sp., a cold-adapted bacterium isolated from marine sediments collected in the South Shetland Islands (Antarctica), is a high molecular weight heteropolysaccharide (>2 × 10^6^ Da) composed mainly of glucose, galactose, and fucose with small amounts of uronic acid and amino acids [27]. EPS ID1 confers significant cryoprotection for the cold-adapted producer bacteria and for the non-adapted ones, which implies that it could be used as an agent for cell cryopreservation, alone or in combination with other CPAs. Recently, we assessed for the first time whether the addition of EPS ID1 to the vitrification/warming media could offer cryoprotection to in vitro matured bovine oocytes [26]. The addition of EPS ID1 at 10 µg/mL to the vitrification/warming media protected bovine oocytes from cryodamage by preserving spindle/chromosome dynamics during vitrification and enhanced embryo development after warming. As far as we know, however, no previous study has investigated the use of exopolysaccharides during embryo cryopreservation.

Embryos must be selected for cryopreservation based on their quality and development stage. Better embryo survival has been related to a more developed embryonic stage on any given day of in vitro culture. Furthermore, differences in survival and pregnancy rates have been reported following cryopreservation of blastocysts obtained at different days (day7 vs. day 8) of in-vitro culture (reviewed by [3]). Moreover, the sexual maturity of the donor has a direct impact on oocyte and embryo competence, with a clear advantage of cow oocytes over those of heifers in terms of embryo production efficiency, under both in-vivo and in-vitro conditions [28].

Our working hypothesis was that EPS ID1 might protect in vitro-cultured blastocysts from cold injury during vitrification and thus enhance embryo development after warming. The present study was therefore designed to examine the effects of adding EPS ID1 to the vitrification solutions on day 7 (D7)- and day 8 (D8)-expanded blastocysts derived from both cow and calf oocytes. Outcomes were assessed in warmed embryos in terms of re-expansion and hatching rates, differential cell counts, apoptosis rate, and relative abundances of mRNAs of genes with a role in apoptosis, oxidative stress, water channels, and gap junctions.

## 2. Results

*Experiment 1. Effect of the addition of EPS to vitrification media on re-expansion and hatching rates of D7 and D8 IVP cow-derived and calf-derived embryos.*

Post-warming re-expansion and hatching rates of blastocysts from D7- and D8-expanded vitrified/warmed blastocysts are shown in Table 1 (cow-derived blastocysts) and Table 2 (calf-derived blastocysts).

All vitrified groups showed significantly lower re-expansion rates at 3 h and 24 h post-warming than the fresh control group, regardless of the treatment, time of culture, or source of oocytes. At 3 h post-warming, no significant differences were observed in re-expansion rates among vitrification treatments. At 24 h post-warming, blastocysts of the VIT-EPS100 group produced higher re-expansion rates (*p* ≤ 0.05) to those of the VIT-Control and VIT-EPS10 groups for both D7 and D8 vitrified/warmed blastocysts derived from cow or calf oocytes. When hatching rates were assessed in vitrified/warmed D7 and D8 cow-derived blastocysts, the addition of 100 µg/mL EPS ID1 to the media produced similar percentages of hatchability to the control fresh group, whereas lower hatching rates (*p* ≤ 0.05) were observed in the VIT-Control and D7 VIT-EPS10 groups. Besides, we observed a higher 24-h re-expansion rate of cow-derived D7 over D8, with no influence from the vitrification process.

D7 blastocysts derived from calf oocytes from the VIT-EPS100 showed a higher hatching yield (*p* ≤ 0.05) compared to the VIT-Control and VIT-EPS10, but lower than the fresh non-vitrified group. Contrarily, hatching rates of calf-derived D8 blastocysts from the VIT-EPS100 group were similar to those of the control fresh group and greater (*p* < 0.05) than those of the VIT-Control and VIT-EPS10.

When blastocysts derived from calf and cow oocytes were compared, D7 blastocysts derived from cow oocytes showed higher re-expansion rates than D7 calf-derived blastocysts both at 3 h and 24 h post-warming, regardless of the vitrification treatment. There was no difference in hatching ability between blastocysts derived from calf and cow oocytes regardless of vitrification treatment or culture length.

*Experiment 2. Effect of the addition of EPS ID1 to vitrification media in TCN, ICM cell number, TE cell number, and apoptosis rate of blastocysts at 24 h post-warming*

Results on the TCN, number of cells in the ICM and TE, and apoptosis rate at 24 h post-warming of surviving expanded and hatched blastocysts from D7- and D8-expanded blastocysts vitrified using different EPS ID1 treatments are shown in Table 3 (cow-derived blastocysts) and Table 4 (calf-derived blastocysts).

There were no differences in TCN, ICM, and TE cell number between treatment groups in either expanded or hatched blastocysts derived from D7 or D8 blastocysts, regardless of the source of oocytes (calf vs. cow). EPS100 surviving expanded and hatched blastocysts had a significantly lower number of apoptotic nuclei than the EPS0 and EPS10 groups, but a larger number than the fresh control group, regardless of the culture length (D7/D8), or source of oocytes (cow/calf). At 24 h post-warming, TCN, ICM, and TE cell counts were higher (*p* ≤ 0.05) in hatched blastocysts than in expanded blastocysts, regardless of the treatment, length of culture, or oocyte source. Moreover, hatched blastocysts had a significantly lower apoptosis rate than expanded blastocysts, regardless of the vitrification treatment, days in culture, or oocyte source.

*Experiment 3: Effect of the addition of EPS ID1 to vitrification media in gene expression of surviving blastocysts at 24 h post-warming.*

**Figure 1:**
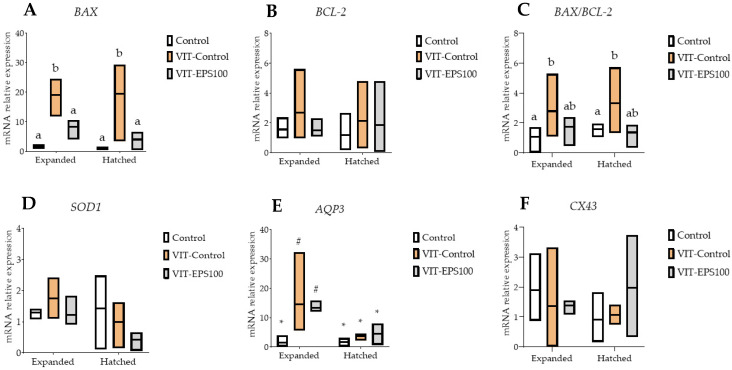
Box plots (solid line indicates the mean) showing expression levels of (**A**) *BAX*, (**B**) *BCL-2*, (**C**) *BAX/BCL-2* ratio, (**D**) *SOD1*, (**E**) *APQ3*, (**F**) *CX43* in post-warmed expanded and hatched blastocysts developed from vitrified/warmed Day 7 expanded blastocysts derived from cow oocytes. Control: fresh non-vitrified expanded blastocysts; VIT-Control: blastocysts vitrified/warmed without EPS ID1 supplementation; VIT-EPS10: blastocysts vitrified/warmed with 10 µg/mL EPS ID1 supplementation; VIT-EPS100: blastocysts vitrified/warmed with 100 µg/mL EPS ID1 supplementation. ^a,b^ Different letters indicate significant differences between treatments (*p* ≤ 0.05).*^,#^ Different symbols indicate differences between developmental stages inside each specific treatment (*p* ≤ 0.05). *BAX*, BCL2-associated X apoptosis regulator; *BCL-2*, BCL2 like 1; *SOD1*, superoxide dismutase 1; *AQP3*, aquaporin 3; *CX43*, connexin 43; Expanded, expanded blastocysts; Hatched, hatching/hatched blastocysts.

To determine whether changes in the expression of developmentally important genes were associated with the addition of EPS ID1 to the vitrification media, the levels of *BAX, BCL2, SOD1, AQP3,* and *CX43* mRNAs in post-warmed surviving expanded and hatched blastocysts were evaluated (Figure 1). As previously observed, the addition of 100 µg/mL EPS to the vitrification media of D7 blastocysts derived from cow oocytes resulted in better survival outcomes at 24 h post-warming than D8 blastocysts or D7 or D8 blastocysts derived from calf oocytes. Because of these findings, the effects of adding EPS to vitrification media on the relative abundance of genes were assessed in expanded and hatched blastocysts derived from D7 cow blastocysts vitrified/warmed in media supplemented with 0 or 100 µg/mL EPS. Non-vitrified expanded and hatched blastocysts served as controls.

No significant differences were detected in relative mRNA abundances for *BCL-2, SOD1,* and *CX43*, regardless of the treatment or embryo stages. When compared to non-vitrified fresh blastocysts, the expression of the *BAX* gene in expanded or hatched blastocysts from the VIT-EPS100 groups remained similar. Expanded and hatched blastocysts derived from blastocysts vitrified/warmed without EPS supplementation exhibited significantly higher *BAX* gene expression than the fresh control or EPS100 groups. The *BAX/BCL-2* ratio was significantly higher in expanded and hatched VIT-Control blastocysts than in fresh non-vitrified blastocysts, but no differences were seen in the VIT-EPS100 group. While there were no differences in gene expression between expanded and hatched blastocysts for *BAX, BCL-2, SOD1,* and *CX43,* the relative abundance of *AQP3* mRNA transcript was higher in expanded blastocysts from the VIT-Control and VIT-EPS100 groups as compared to their hatched counterparts. Although not significant, a considerable trend (*p* = 0.07) of downregulation of the *SOD1* gene was identified in hatched blastocysts when compared to expanded blastocysts, regardless of the treatment.

## 3. Discussion

The present study aimed to assess the cryoprotectant role of EPS ID1 in the vitrification of IVP D7 and D8 bovine embryos derived from cow and calf oocytes. First, we designed a study to determine the optimal concentration of EPS ID1 for its use as a non-permeable cryoprotectant for vitrification of embryos, taking into account the dose-response relationship of its protective effect [27], and our previous results on the vitrification and warming of bovine in vitro-matured oocytes [26].

According to the present findings, the addition of 100 µg/mL of EPS ID1 to vitrification media improves the post-warming survival and hatching ability of blastocysts vitrified/warmed on D7 or D8, in blastocysts derived from cow or calf oocytes. Furthermore, the hatching rate of blastocysts vitrified/warmed with 100 µg/mL EPS ID1 was comparable to that of fresh non-vitrified blastocysts, except for calf blastocysts vitrified/warmed on D7. These findings support our initial hypothesis that an adequate EPS ID1 supplementation could benefit IVP bovine embryo cryopreservation, resulting in enhanced cryotolerance compared to the control vitrification method. Lower concentrations of EPS ID1 (10 µg/mL EPS ID1), on the other hand, resulted in blastocyst re-expansion and hatching rates comparable to vitrification without EPS ID1 supplementation. Based on the previous studies in bovine oocyte vitrification, we can assume that lower concentrations of EPS ID1 are insufficient to exhibit a cryoprotective effect [26]. When a dose-response was examined to improve cryosurvival of in vitro-matured bovine oocytes, 10 µg/mL of EPS ID1 added to the vitrification media resulted in better survival outcomes. Low concentrations of EPS ID1 (1 µg/mL) during oocyte vitrification had no effect on oocyte survival while higher concentrations (1000 µg/mL) were found to be toxic [26]. In the present study, a concentration of 10 µg/mL did not have any positive effect on cryosurvival of blastocysts, a multicellular organism, and a 10 times higher concentration of EPS ID1 was required to notice its protective role.

Avoiding ice crystal formation during vitrification of embryos, whether intracellular or extracellular, is critical to ensure their competence after warming. Hence, a combination of permeable and non-permeable cryoprotectants is incorporated into vitrification/warming solutions. Molecules of biological origin playing a cryoprotective role similar to non-permeating CPAs can be found in nature. Proteins (AFPs), glycoproteins (AFGPs), and carbohydrates (EPS) produced by several species allow them to survive at freezing temperatures by limiting ice recrystallization and stabilizing cell membranes [7,22,29]. Although the composition and antifreeze properties of EPS ID1 have been described [27], the exact mechanism through which EPS ID1 increases embryo cryosurvival remains to be elucidated. In the chemical and spectroscopic analyses of a capsular polysaccharide (CPS) isolated from *Colwellia psychrerythraea* 34H, a unique structure among bacterial EPS was revealed [23]. It consisted of repeating units of a linear tetrasaccharide containing two amino sugars and two uronic acids, decorated by a threonine. While the presence of amino acids is quite uncommon in compounds secreted by bacteria, amino acid motifs are usually found in AFPs structure and are crucial for their interaction with ice [29]. Driven by the similarities between their polysaccharide and AFGPs, Carillo and collaborators [23] examined the ice recrystallization inhibitionactivity of the *Colwellia* CPS and found that ice-binding patterns of the CPS resembled those of AFPs, which immobilize ice grain boundaries and exert a cryoprotective effect. Although its exact primary and secondary structure is unknown, the EPS ID1 composition reveals the presence of monosaccharides and uronic acids decorated by amino acids [27]. As a result, we can speculate that EPS ID1 confers cryoprotection on IVP blastocysts by suppressing ice recrystallization activity.

Arcarons et al. [26] already reported that supplementation of vitrification media with 10 mg/mL EPS ID1 helped to stabilise spindle morphology and improved embryo development in vitrified–warmed oocytes. To our knowledge, this is the first time that a bacterial exopolysaccharide is used as a non-permeable cryoprotectant in the vitrification of embryos. However, various attempts have been made to add biological molecules with protective effects against cold injuries, such as AFPs or AFGPs, to the vitrification solutions of gametes and embryos (for review, see [7]). When 1 mM antifreeze glycoprotein 8 was added to the vitrification media, Liang et al. [30] found that bovine-expanded blastocysts had higher re-expansion rates 12 h after warming compared to the untreated group. Moreover, the addition of 10 mg/mL recombinant fish antifreeze protein (nfeAFP11) combined with controlled warming kept bovine IVD embryos alive for 10 days at 4 °C. In sheep, the addition of 10 μg/mL antifreeze protein from *Anatolica polita* (ApAFP914) to the vitrification media increased the hatching rate at 24 h post-warming but had no effect on embryo survival [14]. In addition, 500 ng/mL of AFPIII enhanced embryo survival after vitrification in rabbits, but 1000 ng/mL decreased it [31]. Other studies found no effects of AFP supplementation in horse [32], sheep [33], mouse [13], and mouse and pig [34] embryos. These findings emphasize an important point that has been mentioned earlier and that must be considered while employing these molecules: their cryoprotective effect relies on concentration, chemical nature, cryopreservation protocol, and features of the biological material [35].

Improved cryosurvival has been associated with more advanced embryonic stages on a given in vitro culture day [36]. In concordance with previous studies conducted in our laboratory [36,37], D7 blastocysts derived from cow oocytes had a significantly higher re-expansion rate than blastocysts vitrified/warmed after 8 days in culture, regardless of the vitrification treatment. In bovine species, early cleavage is associated with better embryo quality and implantation [38,39,40], while the timing of blastocyst formation has been correlated to the in-vitro developmental potential of embryos, assessed by total cell number [39], expression of genes related to embryo quality [41], and pregnancy rates [42]. When blastocysts are cryopreserved, differences in embryo quality become more apparent, with blastocysts vitrified/warmed after 7 days in culture having higher survival and pregnancy rates than blastocysts vitrified/warmed after 8 days in culture [42,43,44,45,46].

In this study, blastocysts derived from cow oocytes vitrified at D7 had a higher re-expansion rate than D7 blastocysts derived from calf oocytes. Oocyte origin along with many other factors such as donor nutrition, environment, stress level, and health could have a great impact on oocyte and embryo competence. The compromised developmental competence of oocytes derived from prepubertal cattle compared to their adult counterparts is well documented in the literature. Incomplete or delayed ooplasmic maturation [47], compromise of developmental competence acquisition [48], altered DNA methylation and mRNA expression profiles [49], and impaired apoptosis regulation [50] can underlay the reduced developmental competence observed in prepubertal oocytes used in in-vitro procedures. Reduced oocyte competence in bovine prepubertal donors results in inferior embryo development [48], blastocyst production, embryo quality [51], and pregnancy rates [52] compared to adult counterparts. It is worth noting that blastocysts derived from calf oocytes vitrified on D8 had similar re-expansion and hatching rates as blastocysts derived from cow oocytes vitrified on D7. This finding could be explained by the fact that calf blastocysts develop more slowly in vitro or have higher embryo quality on D8. As a result, the age of the donor and the speed of blastocyst development appear to be crucial determinants in the vitrification of IVP bovine embryos [36].

Vitrification/warming has been found to have a negative effect on TCN and TE cell numbers [37] and ICM cell number [53,54]. When vitrification media were supplemented with AFPs, Liang et al. [30] found a significantly higher TCN in blastocysts vitrified/warmed with AFGP8 supplementation than in the non-supplemented group. In this study, there were no differences in TCN and the ICM, and TE cell number among vitrification treatments or when compared to the fresh control. Besides, there was no effect of the oocyte source (cow or heifer) or in-vitro culture length (D7 or D8) on total and differential (ICM/TE) cell counts. We hypothesize that our laboratory’s standard vitrification-warming procedure weeds out blastocysts with poor developmental competence, resulting in TCN, ICM, and TE cell numbers equal to fresh blastocysts in the surviving embryos after vitrification-warming. This conjecture is based on the fact that the vitrification-warming procedure involves shrinkage and re-expansion of the blastocoelic cavity. Only blastocysts that re-expanded the blastocoelic cavity after vitrification-warming were considered to have survived, while poor quality blastocysts with inherently low developmental competence, apparently unable to re-expand after vitrification-warming, were removed [55].

While apoptotic events may occur in the embryo to regulate the equilibrium between cell proliferation and dead or eliminate compromised cells [56,57], a correlation between high rates of apoptosis and reduced developmental competence has been found in IVP blastocysts [57,58]. When AFGP8 was added to vitrification media, the incidence of apoptosis in post-warmed AFGP8-treated blastocysts was significantly lower than in the untreated group. However, the proportion of apoptotic cells in vitrified/warmed groups was higher than in fresh blastocysts [30]. These findings are in concordance with our results, where blastocysts vitrified/warmed with 100 µg/mL EPS ID1 showed the lowest number of apoptotic nuclei between vitrification groups, but apoptosis levels were higher in all vitrified/warmed blastocysts when compared to fresh non-vitrified embryos. It is worth noting that the VIT-EPS10 group had the largest number of apoptotic cells among the vitrification groups. Results observed when AFPs are used as cryoprotectants in hypothermic and cryogenic storage indicate a dual action: protective and cytotoxic. AFPs are thought to interact not only with ice crystals but also with cell surfaces and other solutes present during cryopreservation, possibly increasing ice formation and causing cell damage [35]. Although this could be the mechanism via which low doses of EPS ID1 have no effect on embryo development or increase the rate of apoptosis, more research into the mechanism of EPS is required to corroborate this possibility.

Based on results obtained in Experiments 1 and 2, we decided to further examine the effects of EPS1 ID1 addition on gene expression of surviving expanded and hatched blastocysts derived from vitrified/warmed D7 expanded blastocysts. Analysis of apoptosis-related genes showed a higher expression of proapoptotic *BAX* gene and an increased *BAX/BCL-2* ratio in expanded and hatched blastocysts derived from blastocysts vitrified/warmed without EPS ID1 supplementation, whereas supplementation with 100 µg/mL EPS ID1 returned *BAX* levels similar to those seen in fresh control embryos. Previous research revealed that morphologically poor quality or fragmented embryos have greater *BAX* expression levels [59], whereas the opposite occurs in their high-quality counterparts. This suggests that EPS ID1 may have helped to improve embryo quality after vitrification. Similarly, a larger abundance of *BAX* transcripts and lower expression of antiapoptotic *BCL-2* gene were observed in the untreated group compared to blastocysts vitrified/warmed with AFGP8 supplementation [30]. Interestingly, a trend to higher expression of *SOD1* and significantly higher expression of *AQP3* transcripts was observed in surviving expanded blastocysts compared to those able to hatch after 24 h of culture post-warming. The increase observed in *AQP3* expression in expanded blastocysts may reflect the embryo attempts to maximise blastocoel re-expansion after warming in circumstances where the primary mechanism of Na^+^/K^+^-ATPase has been compromised due to cryodamage and mitochondrial impairment [60]. Overall, AQPs appear to play a more prominent role when there is an extremely high rate of fluid transport.

In conclusion, this is the first study investigating the protective effects of different EPS ID1 concentrations on vitrified/warmed bovine blastocysts. The addition of 100 µg/mL of EPS ID1 to the vitrification media increased the post-warming re-expansion of D7 expanded blastocysts derived from both cow and calf oocytes. Except for calf-derived blastocysts vitrified/warmed on D7, the hatching rate of blastocysts vitrified/warmed with 100 µg/mL EPS ID1 was similar to that of fresh blastocysts. After warming, the addition of 100 µg/mL of EPS ID1 to vitrification media reduced apoptosis levels, as measured by cell DNA fragmentation and mRNA levels. Although further research is needed to determine the exact mechanisms underlying the cryoprotective action of EPS ID1, supplementing vitrification media with EPS ID1 should be considered to improve bovine blastocyst cryopreservation techniques. Additional experiments to evaluate the implantation potential of EPS ID1 cryopreserved blastocysts are warranted.

## 4. Materials and Methods

### 4.1. Chemicals and Suppliers

All chemicals and reagents were purchased from Sigma-Aldrich (Merck, Sant Louis, MI, USA) except stated otherwise.

### 4.2. In Vitro Embryo Production

The whole processes of bovine embryo IVP (in-vitro maturation (IVM), in-vitro fertilization (IVF), and in-vitro culture (IVC)) were as previously described elsewhere [4], with some modifications. Briefly, ovaries from cows (>24 months of age) and prepubertal calves (9–12 months of age) were transported from a local abattoir (Escorxador Sabadell S.A., Sabadell, Spain) to the laboratory in saline solution (0.9% NaCl in distilled water) at 35–37 °C. Cumulus–oocyte complexes (COCs) were obtained by aspiration using an 18 g needle from follicles measuring 3–8 mm in diameter. COCs from cow ovaries (*n* = 1497; 7 replicates) and prepubertal ovaries (*n* = 1147; 6 replicates) with a homogeneous cytoplasm and three or more layers of cumulus cells were selected for IVM. After washing (×3) in modified PBS (PBS supplemented with 36 mg/mL pyruvate, 50 mg/mL gentamicin, and 0.5 mg/mL bovine serum albumin (BSA)), selected COCs were placed in 500 μL-well of IVM medium in groups of 40–50 COCs and cultured at 38.5 °C in a 5% CO_2_ humid atmosphere for 24 h. The IVM medium composition was tissue culture medium 199 (TCM-199) enriched with 10% (*v*/*v*) fetal bovine serum (FBS), 10 ng/mL epidermal growth factor, and 50 mg/mL gentamicin.

For IVF, in vitro-matured COCs were transferred in groups of 40–50 to a 250 µL well of IVF medium consisting of 25 mM sodium bicarbonate, 22 mM Na-lactate, 1 mM Na-pyruvate, 6 mg/mL fatty acid-free BSA, and 10 mg/mL heparin–sodium salt. For the obtention of good morphology and high motility spermatozoa, frozen-thawed sperm from a fertile Asturian bull (ASEAVA, Abarrio, Spain) were centrifuged for 10 min at 300× *g* through a discontinuous density gradient (1 mL of 40% and 1 mL of 80% BoviPure diluted in Bovidilute (Nidacon International AB, Göthenburg, Sweden)), resuspended in 3 mL of Boviwash (Nidacon International AB, Göthenburg, Sweden), and pelleted by centrifuging for 5 min at 300× *g*. Once counted in a Neubauer chamber, the resulting spermatozoa were diluted in an adequate volume of IVF medium to have a concentration of 2 × 10^6^ spermatozoa/mL. Then, COCs previously placed in 4-well dishes were inseminated by adding 250 mL of this suspension (final concentration: 1 × 10^6^ spermatozoa/mL) and co-incubated for 18 h at 38.5 °C in a 5% CO_2_ humidified atmosphere.

Eighteen hours post-insemination (hpi), presumptive zygotes were washed and pipetted in PBS to remove cumulus cells. Partially denuded zygotes were placed in 25-μL drops (one embryo per µL) of synthetic oviductal fluid (SOF) (Caisson Labs, UT, USA) medium supplemented with 88.6 μg/mL sodium pyruvate, 2% (*v*/*v*) non-essential amino acids, 1% (*v*/*v*) essential amino acids, 0.96 μg/mL BSA, 2% (*v*/*v*) FBS, and 0.5% gentamicin covered by 3.5–4 mL of Nidoil (Nidacon International AB, Göthenburg, Sweden) for 7 or 8 days at 38.5 °C in a 5% CO_2_, 5% O_2_ humidified atmosphere. D7 blastocyst rate was 32.4 ± 7.9%, and D8 blastocyst rate was 38.6 ± 8.5% for oocytes from adult ovaries, while D7 blastocyst rate was 20.9 ± 2.3% and D8 blastocyst rate was 25.89 ± 5.4% for oocytes from prepubertal ovaries. D7 and D8 grade 1 expanded blastocysts [61] were randomly assigned to the experimental treatments (cow: 7 replicates; calf: 6 replicates).

### 4.3. Supplementation with EPS ID1

The procedure for the EPS ID1 production was described in detail elsewhere [27]. In brief, *Pseudomonas* sp. ID1 were cultured in MM1 minimal medium for 120 h at 11 °C and centrifuged (40,000× *g*, 25 min, 4 °C) to recover the EPS. The supernatant free of cells was stored and pellets were washed three times in Ringer’s solution (Scarlab S.L, Barcelona, Spain) and centrifuged at 40,000× *g* for 20 min at 4 °C to remove any EPS ID1 adhered to the cell surface. Washed supernatants were pooled with the first stored culture supernatant and then subjected to a tangential flow filtration process through 0.22-µm membranes. The filtrate was dialyzed with sterile distilled water 1:10 (*v*/*v*) through 10,000-Da membranes to remove salts, pigments, and other components of the culture medium, and obtain the EPS ID1 in a concentrated and purified form. Finally, the purified EPS ID1 was freeze-dried and stored in hermetically sealed flasks in a fresh and dry place. The working solution was prepared by adding 100 mg of EPS ID1 to 10 mL of HEPES-buffered TCM-199 (10 mg/mL) at 37 °C and mixing vigorously. Aliquots of EPS ID1 working solution were stored at −20 °C until further use.

### 4.4. Embryo Vitrification and Warming

Vitrification was performed with D7 and D8 Grade 1-expanded blastocysts derived from prepubertal and adult oocytes. The short equilibration protocol of the cryotop method [62] was followed with some modifications. Grade 1-expanded blastocysts were selected, washed (3×) with holding medium (HM: HEPES-buffered TCM-199 containing 20% FBS), and randomly allocated to each study group: vitrified without supplementation (EPS0), or in vitrification media supplemented with 10 µg/mL (EPS10) or 100 µg/mL (EPS100) of EPS ID1. Control blastocysts remained as such and were cultured in SOF for 24 additional hours. All steps were performed under a laminar flow hood heated to 38.5 °C and using a stereomicroscope to visualize each step.

#### 4.4.1. Vitrification Protocol

Blastocysts were placed in equilibration solution (ES), consisting of 7.5% (*v*/*v*) ethylene glycol (EG) and 7.5% (*v*/*v*) dimethyl sulphoxide (DMSO) in HM, for 3 min. Then, blastocysts were moved to the vitrification solution (VS) containing 15% (*v*/*v*) EG, 15% (*v*/*v*) DMSO, and 0.5 M sucrose in HM, for 30–40 s. Immediately after, blastocysts (up to two) were loaded onto the cryotop and almost all the solution was removed to leave only a thin layer of the solution covering the blastocysts. The cryotop was plunged into liquid nitrogen within the following 20 s and covered with a plastic protective straw. The entire process from immersion in vs. to plunging into liquid nitrogen was completed within 1 min. The loaded devices were stored in liquid nitrogen.

#### 4.4.2. Warming Protocol

Blastocysts were warmed by quickly immersing the cryotop tip in HM supplemented with 1 M sucrose for 1 min. Then, blastocysts were transferred and incubated in HM supplemented with 0.5 M sucrose for 3 min and then in HM for 5 min. Once vitrified/warmed, blastocysts were cultured in SOF at 38.5 °C in a 5% CO_2_, 5% O_2_ humid atmosphere for 24 h. Vitrified/warmed blastocysts were assessed by morphological evaluation under a stereomicroscope. The survival of vitrified blastocysts was determined as re-expansion rates (proportion of blastocysts that were able to re-expand and/or hatch from the total number of warmed blastocysts) after 3 h and 24 h of recovery in SOF medium, while hatching rates (proportion of hatching/hatched blastocysts from the total number of warmed blastocysts) were assessed at 24 h post-warming (cow: 7 replicates; calf: 6 replicates).

Surviving blastocysts from groups D7 Control, D7 VIT-Control, D7 VIT-EPS10, D7 VIT-EPS100, D8 Control, D8 VIT-Control, D8 VIT-EPS10, and D8 VIT-EPS100 were fixed and immunostained to assess their Total Cell Number (TCN), Inner Cell Mass (ICM) cell number, Trophectoderm cell number (TE), and apoptosis rate (AR) (cow: 4 replicates; calf: 6 replicates).

For gene expression and based on previous results, groups (up to 5) of surviving blastocysts derived from cow oocytes of groups D7 VIT-Control, D7 VIT-EPS0, and D7 VIT-EPS100 were snap-frozen in liquid nitrogen and stored at −80 °C until RNA extraction and RT-qPCR analysis (cow: 3 replicates).

### 4.5. Immunostaining for Differential Cell Count and DNA Fragmentation

**Figure 2:**
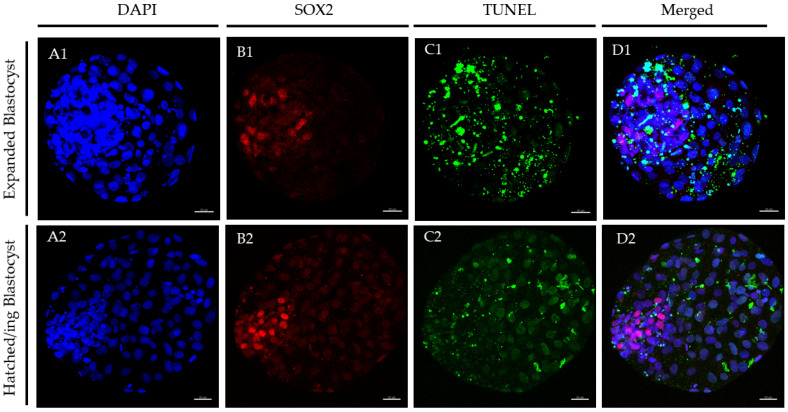
Representative images of post-warmed expanded and hatched blastocysts derived from cow-derived D7 expanded blastocysts vitrified/warmed with vitrification media supplemented with 100 µg/mL EPS ID1. DAPI (blue), SOX2 (red), and TUNEL (green) staining were examined using DAPI, SOX2-Alexa Fluor, and FITC filters, respectively, for total (**A1**,**A2**), ICM (**B1**,**B2**), and apoptotic (**C1**,**C2**) cell counts. An overlay is provided in (**D1**,**D2**). (**A1**,**B1**,**C1**,**D1**): Expanded blastocyst; (**A2**,**B2**,**C2**,**D2**): Hatched blastocyst. Scale bar = 30 μm. DAPI (406-diamidino-2-phenylindole), TUNEL (terminal deoxynucleotidyl transferase dUTP nick end labelling).

At 24 h post-warming, expanded and hatched blastocysts surviving vitrification in each group underwent immunostaining plus the TUNEL (terminal deoxynucleotidyl transferase dUTP nick end labeling) assay to quantify TCN, ICM cell number, TE cell number, and AR [37]. All steps were done at 38.5 °C, unless otherwise stated. Blastocysts were fixed in 2% (*v*/*v*) paraformaldehyde in PBS for 15 min. After fixation, embryos were washed at least three times in PBS and permeabilized in 0.01% Triton X-100 in PBS supplemented with 5% Normal Donkey Serum (NDS) for 1 h at room temperature. Embryos were then washed in PBS for 20 min (×1) and incubated at 4 °C overnight with mouse anti-SOX2 primary antibody (1:100; MA1–014, Invitrogen, Waltham, MA, USA) in a humidified chamber. After washing in 0.005% Triton X-100 in PBS supplemented with 0.05% NDS (PBS-NDS) for 20 min (×3), embryos were incubated with a goat anti-mouse (IgG) secondary antibody Alexa Fluor^TM^ 568 (1:500; A-11004, Thermo Fisher Scientific, Waltham, MA, USA) for 1 h in a humidified chamber. Thereafter, embryos were washed in 0.005% Triton X-100 in PBS-NDS for 20 min (×3) and incubated in the TUNEL reaction mixture, following the manufacturer’s instructions (in situ Cell Death Detection Kit, Fluorescein) for 1 h in the dark. Embryos were then washed thoroughly in PBS for 5 min (×3) and mounted within a 3 µL drop of Vectashield (125 ng mL-1 4′,6-diamidino-2-phenylindole (DAPI)) (Vectorlabs, Burlingame, CA, USA) on coverslips previously treated with poly-L-lysine (1:10) fitted with a self-adhesive reinforcement ring. After flattening the sample with a slide, clear nail varnish was used to seal the preparation, which was stored at 4 °C protected from light until observation within the following 3 days. Positive and negative control samples were included in each assay. As a negative control for SOX2, the primary antibody was omitted. For the TUNEL assay, blastocysts exposed to DNase I for 15 min served as positive controls, and blastocysts not exposed to the terminal TdT enzyme served as negative controls. Confocal images in serial sections separated by 0.38 µm were captured with a confocal laser scanning microscope (Leica TCS SP5, Leica Microsystems CMS GmbH, Mannheim, Germany) at 20× magnification to examine the ICM cell nuclei (SOX2-Alexa Fluor 568™; excitation 562 nm), total cell nuclei (DAPI; excitation 405 nm), and DNA fragmentation (fluorescein isothiocyanate-conjugated TUNEL label; excitation 488 nm). Confocal images were analysed using the Imaris 9.2 software (Oxford Instruments, Oxfordshire, UK). Individual nuclei were counted as intact (TUNEL(−); blue/red stain) or fragmented (TUNEL(+), green stain) DNA; TE cells (SOX2(−); blue stain) or ICM cells (SOX2(+); red stain). The TCN was calculated as the addition of TE and ICM cells. The AR was calculated as the ratio of TUNEL(+) cells/total number of cells. Confocal microscopic images obtained after staining of post-warmed expanded and hatched blastocysts are shown in Figure 2.

### 4.6. Gene Expression Analysis by Reverse Transcription and Quantitative PCR (rt-qPCR)

The procedures used for RNA extraction and real-time reverse transcription-quantitative polymerase chain reaction (RT-qPCR) are described elsewhere [63]. For gene expression analysis, blastocysts in pools up to 5 were washed three times with Dulbecco’s PBS supplemented with polyvinyl alcohol (PVA; 0.01% *w*/*v*) at 38.5 °C and then pipetted within the minimum volume into 1.5 mL Eppendorf tubes. Immediately, the tubes were plunged into liquid nitrogen and stored at −80 °C until further processing.

As the first step of RT-qPCR, poly-(A)-RNA was extracted from blastocyst pools using the Dynabeads mRNA Direct Extraction Kit (Thermo Fisher Scientific, Waltham, MA, USA) according to the manufacturer’s instructions with slight modifications. All steps were performed at RT, except when indicated otherwise. Each pool of blastocysts contained in the 1.5 mL Eppendorf tube was lysed in 50 mL lysis buffer for 5 min with gentle pipetting. After that, the fluid lysate was hybridized with 10 μL of pre-washed beads for 5 min with gentle shaking. After hybridization, poly-(A)-RNA–bead complexes were washed twice in 50 μL Washing Buffer A and two more times in 50 μL Washing Buffer B. Next, samples were eluted in 16 μL Elution Buffer (Tris-HCl) and heated to 70 °C for 5 min. Immediately after extraction, 4 μL qScript cDNAsupermix (Quanta Biosciences, Beverly Hills, CA, USA) were added, and reverse transcription (RT) was performed using oligo-dT primers, random primers, dNTPs, and qScript reverse transcriptase. The RT reaction was run for 5 min at 25 °C, followed by 1 h at 42 °C to allow the reverse transcription of mRNA and for 10 min at 70 °C to denature the reverse transcriptase enzyme. The resulting cDNA was diluted in 25 μL of Tris-HCl (elution solution). The relative abundance of mRNA transcripts was quantified by qPCR using a 7500 Real-Time PCR System (Applied Biosystems, Foster City, CA, USA). The qPCR master mix contained 10 µL of Fast SYBR Green Master Mix (Thermo Fisher Scientific, Waltham, MA, USA), 1.5 µL of each primer (500 nM; Thermo Fisher Scientific, Waltham, MA, USA), and 2 µL cDNA template. DEPC-treated water (Thermo Fisher Scientific, Waltham, MA, USA) was added to a final volume of 20 µL. The PCR amplification consisted of one cycle of denaturation at 95 °C for 10 min, followed by 45 cycles of amplification with a denaturation step at 95 °C for 15 s, an annealing step at 60 °C (the appropriate annealing temperature for the primers) for 1 min, and a final extension step at 72 °C for 40 s. Fluorescence data were acquired during the final extension step. The melting curve of each amplified PCR product was checked and run in gel electrophoresis (2% agarose gel containing 0.1 μg/mL SafeView™ Plus; Applied Biological Materials, Richmond, BC, Canada). Two technical replicates from each of the three biological replicates per individual gene were included in each reaction. Negative controls for the template and the primers were also included and amplified by PCR to ensure no cross contamination.

The relative expression of five candidate genes (*BAX, BCL-2, AQP3, SOD1,* and *CX43*) in vitrified/warmed surviving blastocysts was quantified using the comparative threshold cycle (Ct) method. The threshold cycle for each sample, set in the log-linear phase and indicator of the PCR cycle number at which the fluorescence generated was just above background fluorescence, was determined from fluorescence data acquired after each elongation step. Within this amplification curve region, a difference of one cycle is equivalent to the doubling of the amplified PCR product. To calculate ΔCt values, the housekeeping (HK) genes *PPIA* and *H3F3A* were used as normalizers. Thus, the mean PPIA and H3F3A Ct values for each sample were subtracted from the Ct value separately for each replicate and each target gene. The ΔCt value was subtracted from the average ΔCt value for all embryos per each target gene and each stage to calculate ΔΔCt. To calculate the fold differences in relative transcript abundances, the 2^−(ΔΔCt)^ formula was used assuming an amplification efficiency of 100%. Negative controls for template and primers were not amplified or returned as Ct 10 points higher than the average Ct for the genes amplified in samples. The analysis was repeated independently four times. Primer sequences, GenBank accession numbers, and amplicon sizes are indicated in Table 5.

### 4.7. Statistical Analysis

To perform all statistical tests, the statistical package SPSS Version 25.0 (IBM, Armonk, NY, USA) for Windows was used. The data were first checked for normality using the Shapiro–Wilk’s test and for homogeneity of variances using the Levene test.

Re-expansion and hatching rates were compared by two-way analysis of variance (ANOVA) followed by Bonferroni test for pair-wise comparisons. Total cell count, number of cells in ICM, and apoptotic index were analysed by a three-factor general linear model. Relative transcript abundances were evaluated by ANOVA followed by the post-hoc Bonferroni test. If data was not normally distributed or variances were not homogenous, a linear transformation into arcsine square roots, square roots, or logarithms was conducted. When transformed data did not fulfil parametric assumptions, Kruskal–Wallis and Mann–Whitney tests were used as non-parametric alternatives.

The mean ± standard error of the mean (SEM) is used to express data. Significance was set at *p* ≤ 0.05.

## Figures and Tables

**Table 1:** Post-warming re-expansion and hatching rates of cow-derived D7 and D8 expanded blastocysts after vitrification with VIT-Control, VIT-EPS10, and VIT-EPS100.

Blastocyst Derived from Cow Oocytes								
	Day 7 Blastocysts				Day 8 Blastocysts			
	*n*	Post-Warming			*n*	Post-Warming		
		Re-Expansion Rate (%) (3 h)	Re-Expansion Rate (%) (24 h)	Hatching Rate (%) (24 h)		Re-Expansion Rate (%) (3 h)	Re-Expansion Rate (%) (24 h)	Hatching Rate (%) (24 h)
Control	101	100 ^a^	100 ^a^	34.2 ± 1.7 ^a^	40	100 ^a^	100 ^a^	52.5 ± 8.5
VIT-Control	97	34.7 ± 2.4 ^b^	74.9 ± 3.3 ^b,^*	22.7 ± 5.7 ^b^	40	39.5 ± 8.5 ^b^	52.1 ± 4.1 ^b,#^	32.5 ± 2.5 ^b^
VIT-EPS10	40	50.0 ± 7.1 ^b^	70.0 ± 2.4 ^b,^*	20.0 ± 5.7 ^b^	45	35.0 ± 2.9 ^b^	47.9 ± 4.3 ^b,#^	28.2 ± 5.4 ^b^
VIT-EPS100	92	49.1 ± 3.7 ^b^	86.4 ± 3.7 ^c,^*	46.5 ± 5.1 ^a^	30	46.7 ± 6.6 ^b^	66.7 ± 5.7 ^c,#^	56.7 ± 8.8 ^a^

Data are shown as mean ± SEM. ^a,b,c^ Values within columns with different superscripts indicate significant differences between treatments (*p* ≤ 0.05); *^,#^ Values within rows with different superscripts indicate significant differences between culture lengths (Day 7 and Day 8) (*p* ≤ 0.05). Re-expansion rate: proportion of blastocysts that were able to re-expand and/or hatch from the total number of warmed blastocysts; Hatching rate: proportion of hatching/hatched blastocysts from the total number of warmed blastocysts. Control: fresh non-vitrified expanded blastocysts; VIT-Control: blastocysts vitrified/warmed without EPS ID1 supplementation; VIT-EPS10: blastocysts vitrified/warmed with 10 µg/mL EPS ID1 supplementation; VIT-EPS100: blastocysts vitrified/warmed with 100 µg/mL EPS ID1 supplementation.

**Table 2:** Post-warming re-expansion and hatching rates of calf D7 and D8 expanded blastocysts after vitrification with VIT-Control, VIT-EPS10, and VIT-EPS100.

Blastocyst Derived from Calf Oocytes								
	Day 7 Blastocysts				Day 8 Blastocysts			
	*n*	Post-Warming			*n*	Post-Warming		
		Re-Expansion Rate (%) (3 h)	Re-Expansion Rate (%) (24 h)	Hatching Rate (%) (24 h)		Re-Expansion Rate (%) (3 h)	Re-Expansion Rate (%) (24 h)	Hatching Rate (%) (24 h)
Control	34	100 ^a^	100 ^a^	57.5 ± 13.2 ^a^	40	100 ^a^	100 ^a^	52.5 ± 8.5 ^a^
VIT-Control	40	29.5 ± 7.5 ^b^	57.5 ± 4.7 ^b^	22.5 ± 7.5 ^b^	43	32.1 ± 4.6 ^b^	57.3 ± 7.1 ^b^	20.2 ± 4.3 ^b^
VIT-EPS10	38	31.7 ± 2.4 ^b^	55.0 ± 2.8 ^b^	18.7 ± 6.5 ^b^	40	37.5 ± 4.8 ^b^	50.0 ± 4.1 ^b^	15.0 ± 6.4 ^b^
VIT-EPS100	32	34.1 ± 6.7 ^b^	71.5 ± 3.8 ^c^	33.3 ± 12.0 ^c^	30	46.7 ± 8.8 ^b^	70.0 ± 5.7 ^c^	40.0 ± 5.4 ^a^

Data are shown as mean ± SEM. ^a,b,c^ Values within columns with different superscripts indicate significant differences between treatments (*p* ≤ 0.05). Re-expansion rate: proportion of blastocysts that were able to re-expand and/or hatch from the total number of warmed blastocysts; Hatching rate: proportion of hatching/hatched blastocysts from the total number of warmed blastocysts. Control: fresh non-vitrified expanded blastocysts; VIT-Control: blastocysts vitrified/warmed without EPS ID1 supplementation; VIT-EPS10: blastocysts vitrified/warmed with 10 µg/mL EPS ID1 supplementation; VIT-EPS100: blastocysts vitrified/warmed with 100 µg/mL EPS ID1 supplementation.

**Table 3:** Total cell number, number of cells in the ICM and TE, and rate of apoptotic cells of surviving expanded and hatched blastocyst produced from cow-derived blastocysts vitrified/warmed using VIT-Control, VIT-EPS10, and VIT-EPS100 treatments.

Blastocyst Derived from Cow Oocytes									
		Day 7 Blastocysts							
		TCN ± SEM		ICM Cell Number ± SEM		TE Cell Number ± SEM		AR ± SEM	
	*n*	Expanded	Hatched	Expanded	Hatched	Expanded	Hatched	Expanded	Hatched
Control	44	134.6 ± 6.4 ^1^	219.3 ± 4.8 ^2^	32.1 ± 1.4 ^1^	43.6 ± 1.7 ^2^	102.5 ± 5.4 ^1^	175.7 ± 4.9 ^2^	6.9 ± 0.5 ^a,1^	4.5 ± 0.8 ^a,2^
VIT-Control	42	140.1 ± 5.8 ^1^	214.1 ± 2.9 ^2^	22.7 ± 3.2 ^1^	47.5 ± 1.9 ^2^	117.4 ± 14.8 ^1^	166.6 ± 2.2 ^2^	15.6 ± 1.0 ^b,2^	13.1 ± 0.6 ^b,2^
VIT-EPS10	40	123.7 ± 6.4 ^1^	205.6 ± 3.4 ^2^	26.2 ± 1.5 ^1^	42.3 ± 2.4 ^2^	97.5 ± 11.0 ^1^	163.3 ± 10.7 ^2^	18.8 ± 2.3 ^c,2^	16.4 ± 0.8 ^c,2^
VIT-EPS100	36	133.8 ± 8.2 ^1^	224.1 ± 2.4 ^2^	33.6 ± 3.9 ^1^	44.3 ± 2.6 ^2^	100.2 ± 6.5 ^1^	179.8 ± 2.5 ^2^	13.5 ± 1.2 ^d,2^	11.5 ± 0.6 ^d,2^
		**Day 8 blastocysts**							
		**TCN ± SEM**		**ICM cell number ± SEM**		**TE cell number ± SEM**		**AR ± SEM**	
	***n***	**Expanded**	**Hatched**	**Expanded**	**Hatched**	**Expanded**	**Hatched**	**Expanded**	**Hatched**
Control	40	145.0 ± 8.1 ^1^	216.5 ± 3.4 ^2^	41.2 ± 3.4 ^1^	53.7 ± 0.8 ^2^	103.8 ± 6.0 ^1^	207.8 ± 3.6 ^2^	5.3 ± 0.6 ^a,1^	4.7 ± 0.3 ^a,2^
VIT-Control	40	121.8 ± 5.6 ^1^	206.4 ± 5.9 ^2^	27.7 ± 3.2^,1^	49.5 ± 1.2 ^2^	94.1 ± 14.8 ^1^	156.9 ± 2.9 ^2^	13.6 ± 0.5 ^b,1^	11.1 ± 0.9 ^b,2^
VIT-EPS10	45	137.9 ± 10.8 ^1^	203.6 ± 7.8 ^2^	33.2 ± 4.0 ^1^	48.1 ± 3.5 ^2^	104.7 ± 8.3 ^1^	155.5 ± 7.2 ^2^	20.3 ± 1.3 ^c,1^	17.9 ± 1.6 ^c,2^
VIT-EPS100	30	143.0 ± 6.2 ^1^	209.8 ± 4.5 ^2^	38.0 ± 2.9 ^1^	52.3 ± 2.8 ^2^	105.0 ± 12.3 ^1^	157.5 ± 4.6 ^2^	9.2 ± 1.4 ^d,1^	7.8 ± 0.6 ^d,2^

Data are shown as mean ± SEM. ^a,b,c,d^ Values within columns with different superscripts indicate significant differences between treatments (*p* ≤ 0.05); ^1,2^ Values within rows with different superscripts indicate significant differences between stages (Expanded and Hatched) (*p* ≤ 0.05). TCN: Total cell number; ICM: Inner Cell Mass; TE: Trophectoderm; AR: Apoptosis rate. Control: fresh non-vitrified expanded blastocysts; VIT-Control: blastocysts vitrified/warmed without EPS ID1 supplementation; VIT-EPS10: blastocysts vitrified/warmed with 10 µg/mL EPS ID1 supplementation; VIT-EPS100: blastocysts vitrified/warmed with 100 µg/mL EPS ID1 supplementation.

**Table 4:** Total cell number; n° of cells in the ICM and TE; and rate of apoptotic cells of surviving expanded and hatched blastocyst produced from calf-derived blastocysts vitrified/warmed using VIT-Control, VIT-EPS10, and VIT-EPS100 treatments.

Blastocyst Derived from Calf Oocytes									
		Day 7 Blastocysts							
		TCN ± SEM		ICM Cell Number ± SEM		TE Cell Number ± SEM		AR ± SEM	
	*n*	Expanded	Hatched	Expanded	Hatched	Expanded	Hatched	Expanded	Hatched
Control	34	120.5 ± 5.3 ^a,1^	190.2 ± 4.3 ^a,2^	34.5 ± 1.9 ^a,1^	49.1 ± 2.2 ^a,2^	86.0 ± 5.3 ^a,1^	141.1 ± 4.3 ^a,2^	7.2 ± 0.6 ^a,1^	5.2 ± 0.5 ^a,2^
VIT-Control	40	134.4 ± 7.1 ^a,1^	211.3 ± 3.6 ^a,2^	40.3 ± 7.2 ^a,1^	56.7 ± 3.2 ^a,2^	94.1 ± 4.8 ^a,1^	154.6 ± 4.5 ^a,2^	13.6 ± 0.9 ^b,1^	11.2 ± 1.2 ^b,2^
VIT-EPS10	38	138.0 ± 6.4 ^a,1^	206.1 ± 5.8 ^a,2^	33.0 ± 3.7 ^a,1^	47.6 ± 4.0 ^a,2^	105.0 ± 6.1 ^a,1^	158.5 ± 6.0 ^a,2^	17.9 ± 1.2 ^c,1^	14.3 ± 1.5 ^c,2^
		**Day 8 blastocysts**							
		**TCN ± SEM**		**ICM cell number ± SEM**		**TE cell number ± SEM**		**AR ± SEM**	
	***n***	**Expanded**	**Hatched**	**Expanded**	**Hatched**	**Expanded**	**Hatched**	**Expanded**	**Hatched**
Control	40	113.2 ± 6.8 ^a,1^	215.4 ± 2.9 ^a,2^	32.5 ± 1.8 ^a,1^	50.1 ± 1.4 ^a,2^	80.7 ± 5.8 ^a,1^	165.3 ± 3.3 ^a,2^	6.2 ± 0.5 ^a,1^	5.1 ± 0.4 ^a,2^
VIT-Control	43	98.2 ± 5.3 ^a,1^	209.5 ± 1.6 ^a,2^	22.6 ± 0.8 ^a,1^	44.3 ± 2.5 ^a,2^	75.6 ± 2.9 ^a,1^	165.2 ± 8.3 ^a,2^	15.1 ± 1.2 ^b,1^	12.1 ± 0.7 ^b,2^
VIT-EPS10	40	129.8 ± 6.9 ^a,1^	204.2 ± 2.1 ^a,2^	35.3 ± 3.2 ^a,1^	44.5 ± 2.9 ^a,2^	94.5 ± 10.1 ^a,1^	159.7 ± 1.8 ^a,2^	21.6 ± 0.7 ^c,1^	15.4 ± 0.6 ^c,2^
VIT-EPS100	30	120.8 ± 5.7 ^a,1^	213.3 ± 3.1 ^a,2^	32.8 ± 5.3 ^a,1^	53.7 ± 1.6 ^a,2^	88.0 ± 10.1 ^a,1^	159.6 ± 2.7 ^a,2^	10.5 ± 1.5 ^d,1^	8.4 ± 0.5 ^d,2^

Data are shown as mean ± SEM. ^a,b,c,d^ Values within columns with different superscripts indicate significant differences between treatments (*p* ≤ 0.05); ^1,2^ Values within rows with different superscripts indicate significant differences between stages (Expanded and Hatched) (*p* ≤ 0.05). TCN: Total cell number; ICM: Inner Cell Mass; TE: Trophectoderm; AR: Apoptosis rate. Control: fresh non-vitrified expanded blastocysts; VIT-Control: blastocysts vitrified/warmed without EPS ID1 supplementation; VIT-EPS10: blastocysts vitrified/warmed with 10 µg/mL EPS ID1 supplementation; VIT-EPS100: blastocysts vitrified/warmed with 100 µg/mL EPS ID1 supplementation.

**Table 5:** Primers used for reverse transcription-quantitative polymerase chain reaction (rt-qPCR) (NCBI, National Center for Biotechnology Information).

Symbol	Primer Sequences (5′-3′)	Amplicon Size (bp)	GenBank Accession No.
BCL2 associated X apoptosis regulator (*BAX)*	F: ACCAAGAAGCTGAGCGAGTG	116	NM_173894.1
	R: CGGAAAAAGACCTCTCGGGG		
BCL2-like 1 (*BCL-2)*	F: GAGTTCGGAGGGGTCATGTG	211	NM_001166486.1
	R: TGAGCAGTGCCTTCAGAGAC		
Superoxide dismutase 1 *(SOD1)*	F: ACACAAGGCTGTACCAGTGC	102	NM_174615.2
	R: CACATTGCCCAGGTCTCCAA		
Aquaporin 3 *(AQP3)*	F: GTGGACCCCTACAACAACCC	222	NM_001079794.1
	R: CAGGAGCGGAGAGACAATGG		
Connexin 43 (*CX43)*	F:TGGAATGCAAGAGAGGTTGAAGAGG	294	NM_174068.2
	R: AACACTCTCCAGAACACATGATCG		
Peptidylprolyl isomerase A *(PPIA)*	F: CATACAGGTCCTGGCATCTTGTCC	108	NM_178320.2
	R: CACGTGCTTGCCATCCAACC		
H3.3 histone A *(H3F3A)*	F: CATGGCTCGTACAAAGCAGA	136	NM_001014389.2
	R: ACCAGGCCTGTAACGATGAG		

## Data Availability

Data is contained within the article.
